# Progress in EEG-Based Brain Robot Interaction Systems

**DOI:** 10.1155/2017/1742862

**Published:** 2017-04-05

**Authors:** Xiaoqian Mao, Mengfan Li, Wei Li, Linwei Niu, Bin Xian, Ming Zeng, Genshe Chen

**Affiliations:** ^1^School of Electrical Engineering and Automation, Tianjin University, Tianjin 300072, China; ^2^Department of Computer & Electrical Engineering and Computer Science, California State University, Bakersfield, CA 93311, USA; ^3^State Key Laboratory of Robotics, Shenyang Institute of Automation, Shenyang, Liaoning 110016, China; ^4^Department of Math and Computer Science, West Virginia State University, Institute, WV 25112, USA; ^5^Intelligent Fusion Technology, Inc., Germantown, MD 20876, USA

## Abstract

The most popular noninvasive Brain Robot Interaction (BRI) technology uses the electroencephalogram- (EEG-) based Brain Computer Interface (BCI), to serve as an additional communication channel, for robot control via brainwaves. This technology is promising for elderly or disabled patient assistance with daily life. The key issue of a BRI system is to identify human mental activities, by decoding brainwaves, acquired with an EEG device. Compared with other BCI applications, such as word speller, the development of these applications may be more challenging since control of robot systems via brainwaves must consider surrounding environment feedback in real-time, robot mechanical kinematics, and dynamics, as well as robot control architecture and behavior. This article reviews the major techniques needed for developing BRI systems. In this review article, we first briefly introduce the background and development of mind-controlled robot technologies. Second, we discuss the EEG-based brain signal models with respect to generating principles, evoking mechanisms, and experimental paradigms. Subsequently, we review in detail commonly used methods for decoding brain signals, namely, preprocessing, feature extraction, and feature classification, and summarize several typical application examples. Next, we describe a few BRI applications, including wheelchairs, manipulators, drones, and humanoid robots with respect to synchronous and asynchronous BCI-based techniques. Finally, we address some existing problems and challenges with future BRI techniques.

## 1. Introduction

There are several approaches to brain activity measurements, such as magnetoencephalogram (MEG), near infrared spectroscopy (NIRS), electrocorticogram (ECoG), functional magnetic resonance imaging (fMRI), and electroencephalogram (EEG) [1]. Brain machine interface (BMI) [2, 3] or Brain Computer Interface (BCI) [4–6] provides a new nonmuscular channel for sending messages and commands to the external world. A BCI creates an additional communication channel for users who are not able communicate via normal pathways and computers. In BCI systems, the signal acquisition devices are generally divided into two categories: invasive and noninvasive. In an invasive BCI system, arrays of microelectrodes are permanently implanted in the cerebral cortex [7]. The brain signals are recorded from ensembles of single brain cells (also known as single units) or the activity of multiple neurons (also known as multiunits) [8]. Schmidt investigated the possibility of making long-term connections to the central nervous system with microelectrodes to control external devices [9]. In the year 2000, Nicolelis had successfully realized an invasive BMI on a night monkey, which reconstructed its arm movements to obtain food by operating a joystick. This open-loop BMI-based system was upgraded to test a closed-loop motor control on a macaque monkey. The monkey was able to control movements of a robot arm to grasp an object by a moving cursor on a video screen via visual feedback [10]. In terms of human beings, Hochberg et al. demonstrated the ability of two people with long-standing tetraplegia to use a neural interface system to control a robotic arm to perform three-dimensional reach and grasp movements [11]. Participants controlled the arm and hand over a broad space without explicit training, using signals decoded from a small, local population of motor cortex neurons, recorded from a 96-channel microelectrode array. Schwartz et al. comprehensively reviewed invasive BMI technologies for mind-controlled robot systems [12].

An EEG device, as a representative of noninvasive technology, found a wide application in both clinical and research fields [13–16] due to its low cost and portability. Invasive BCI systems are mainly used to restore special sensations, such as visual sense, and motor functions for paralyzed patients. The quality of neural signals is relatively high because the microelectrodes are directly implanted into the cerebral grey matter. However, invasive BCI systems have the disadvantage of easily causing immune reaction and callus, which most likely lead to the regression and disappearance of neural signals.

In order to solve these problems, many researchers have focused on noninvasive BCI systems because of their ease of use, portability, low cost, and low damage to human bodies. Different from the invasive BCI systems, which record the single-unit activity from within cortex, the noninvasive BCI systems use EEGs to record brain electrical activities from the scalp [17]. Therefore, noninvasive BCI systems have found a wider application. Early in the 1990s, Niels Birbaurmer had translated the EEG signals of paralyzed patients into control commands to control the cursor of a computer. In the following years, the EEG-based BCI has been largely researched to analyze the characteristics of brain signals from the scalp and apply it to control intelligent devices to assist paralyzed patients with their daily lives. The typically used signal acquisition devices include a series of products (g.USBamp [18–20], g.BSamp [21, 22], and g.BCIsys [23]) made by g.tec in Austria, Cerebus [24–28] made by BlackRock Microsystems in USA, a series of products with 64, 128, or 256 channels (SynAmps 2 [29–33]) made by Compumedics Neuroscan in Australia, wireless Emotiv EPOC [34–36] made by Emotiv Systems in USA, BrainNet-36 [37], ANT-Neuro [38], FlexComp Infiniti encoder [39], and so forth. And the most commonly used BCI operating system is BCI2000 [40] because it is highly flexible and interchangeable and especially can incorporate alone or in combination with any brain signals, signal processing methods, output devices, and operating protocols.

Based on brain activity patterns, the EEG-based BCI systems are categorized into four different types: event-related desynchronization/synchronization (ERD/ERS) [41], steady-state visual evoked potential (SSVEP) [42], event-related potential (ERP) [43], and slow cortical potential (SCP) [44]. Among them, the SSVEP, ERPs, ERD/ERS, and their hybrids [45–48] attract the interests of researchers.

In the application of BCI-based cognitive models to control external mechanical devices, such as a robot arm [49], a wheelchair [50], or a humanoid robot [34], Brain Robot Interaction (BRI) [24, 51, 52] has become more and more popular. A BRI system is a closed-loop control system that uses brain signals in combination with surrounding information feedback. The collected brain activities must be decoded to generate commands for robots to execute an action or a task that an operator wants to fulfill. The robot must provide feedback of the surroundings to the operator, to assist in making proper decisions. Therefore, an ideal setup for a BRI system usually consists of evoking sources (for SSVEP or ERP) to generate specific brain signals, signal acquisition devices, data analyzing systems, and control objects, among which the signal generating and data analyzing are the most challenging and worthy researching tasks. More and more researchers focus their attention on discovering new evoking mechanisms and testing novel decoding algorithms.

In this paper, we present a comprehensive review and a critical analysis of the three main EEG models with respect to brain signal generation, methods of feature extraction, and feature classification. Then, we list some applications of synchronous and asynchronous BRI systems, especially for humanoid robots. Last, we focus on discussing the challenges and future perspectives of brain signal modeling and the difficulties of BRI.

## 2. EEG-Based Brain Signal Models

### 2.1. SSVEP

#### 2.1.1. Evoking Mechanism

In EEG-based brain signal models, SSVEP is generated by visual stimuli. From retinal photoreceptors, visual percepts propagate first to the visual areas and next to the rest of the brain [53]. Following the presentation of visual stimuli, sensory evoked potentials (SEPs) and termed visually evoked potentials (VEPs) can be recorded in the visual areas. VEPs elicited by brief stimuli are usually transient responses of the visual system. Transient evoked potentials are responses of the system under study to sudden changes (jumps or steps) in the input [54]. About 50 years ago, Regan started experimenting with long stimulus trains, consisting of sinusoidally modulated monochromatic light [55]. These stimuli produced a stable VEP of small amplitude, which could be extracted by averaging over multiple trials. These EEG waves were termed as “steady-state” visually evoked potentials (SSVEP) of the human visual system. SSVEPs can also be found in animals, such as in primates [56] or in cats [57].

SSVEP is a steady-state physical response to outside periodic stimuli and generated at the primary visual cortex without triggering senior visual information process [62]. Since SSVEP is generated at the occipital EEG electrodes (including Oz, O1, and O2 [63]), the corresponding areas have the strongest power. Although the electrodes used in SSVEP vary from person to person, the most reasonable electrodes used in SSVEP mainly include Oz, O1, O2, Pz, P3, P4, and some surrounding electrodes located at the occipital. Researchers had concluded that SSVEP evoking frequencies had a wide range from 1 to at least 90 Hz, and the steady-state potentials exhibited clear resonance phenomena around 10, 20, 40, and 80 Hz [64]. The most commonly used frequencies range from 4 to 60 Hz. In terms of SSVEP evoking, the repetitive visual stimulus (RVS) [58] mainly include simple square flicker, checkerboard, gratings, and light-emitting diode (LED) [65].

#### 2.1.2. Experimental Paradigm

SSVEP-based BCIs allow users to select a target by means of an eye-gaze. The user visually fixes attention on a target and the BCI identifies the target through SSVEP features analysis [172]. Considering a BCI as a communications channel, SSVEP-based BCIs can be classified into three categories depending on the specific stimulus sequence modulation in use [173]: time modulated VEP (t-VEP) BCIs, frequency modulated VEP (f-VEP) BCIs, and pseudorandom code modulated VEP (c-VEP) BCIs. VEPs that react to different stimulus sequences should be orthogonal or near orthogonal to each other in some domain, to ensure reliable identification of the target. In a t-VEP BCI, the flash sequences of different targets are orthogonal in time. That is, the flash sequences for different targets are either strictly nonoverlapping or stochastic. In an f-VEP BCI, each target is flashed at a unique frequency, generating a periodic sequence of evoked responses with the same fundamental frequency as its harmonics. In a c-VEP BCI, pseudorandom sequences are used. The duration of ON and OFF states of each target's flash is determined by a pseudorandom sequence. Signal modulations can optimize the information transfer rate. Indeed, code modulation provides the highest communication speed.

**Figure 1:**
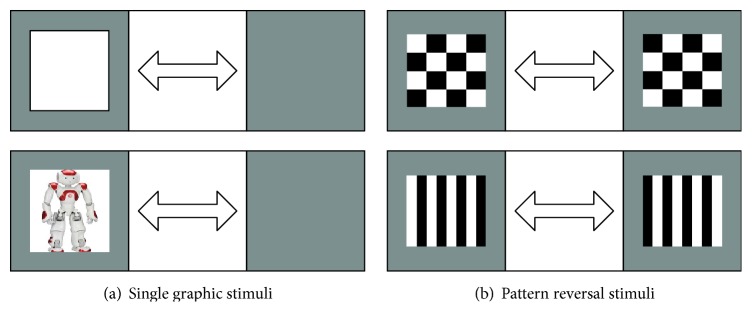
(a) Single graphic stimuli: the graphical object alternately appears and disappears in the background. (b) Pattern reversal stimuli: at least two patterns are alternated at a specified frequency [58].

To elicit an SSVEP, a RVS has to be presented to the user. The RVS can be rendered on a computer screen by alternating graphical patterns or with external light sources able to emit modulated light. Alternating graphical patterns mainly include single graphic and pattern reversal stimuli. Single graphic stimuli could be a rectangle, square, arrow, or robot picture and rendered on a computer screen and appear from and disappear into the background at a specified rate, as shown in Figure 1(a). Pattern reversal stimuli could be a checkerboard or grating that is rendered by oscillatory alternation of graphical patterns, as shown in Figure 1(b). They consist of at least two patterns that are alternated at a specified number of alternations per second. The external light can flash with any frequency and the graphical patterns with only certain frequencies because of the computer screen refresh rate limitations.

### 2.2. ERP

#### 2.2.1. Evoking Mechanism

ERP is generated when a specific stimulus acts on the sensory system of the brain or some mental factor occurs. Subsequently, ERP is evoked in response to the emerging or disappearing of the stimuli. Classical ERPs include several positive and negative waves, such as P1, N1, P2, N2, and P3 (namely, P300) according to the emerging sequences and polarities. The N1 is associated with attention [174] and P2 with stimulus encoding [175]. N2 has been associated with “response selection” or “response activation” [176] and P300 with “context updating” [177] or “context closure” [178]. As the “exogenous component,” the P1, N1, and P2 components are easily influenced by physical stimuli characters, while as the “endogenous component,” N2 and P3 are not influenced by physical stimuli characters.

In 1965, Sutton et al. discovered an electrical potential that exhibited a positive fluctuation within approximately 300 ms after the presentation of an unexpected event (visual, auditory, etc.) [179]. Smith et al. named this potential “P300” potential based on its polarity and relatively fixed latency [180]. A P300 potential is induced prominently in channels Pz, Fz, and Cz in the midline centroparietal regions, and its latency varies from 300 ms to 800 ms when a set of visual stimuli are presented unexpectedly in a random sequence [181]. Therefore, the most commonly used electrodes in ERP are mainly located in the midline centroparietal regions, such as Fz, Cz, Pz, Oz, and their surrounding ones. The P300 component has a relatively high amplitude of 5–20 *μ*V and can be found in EEG after a single stimulus without superposition, so it has wide applications in BRI.

#### 2.2.2. Experimental Paradigm

A P300 system often uses stimuli with different characters, contents, and decoding methods to run the corresponding cognitive process of the subject, according to the different contents and purposes of the research. The visually evoked P300 system often adapts the visual oddball paradigm, in which two different visual stimuli are presented to the subject randomly and the standard stimulus appears generally and the bias stimulus incidentally. The bias stimulus is called target stimulus when the subject reacts to it. The P300 component will be observed in 300 ms after the target stimulus appears [182]. Except the evoking paradigm of presenting a single visual stimulus in turn, researchers have put forward more and more P300 evoking paradigms to present more stimuli efficiently in the P300-based character speller system.

**Figure 2:**
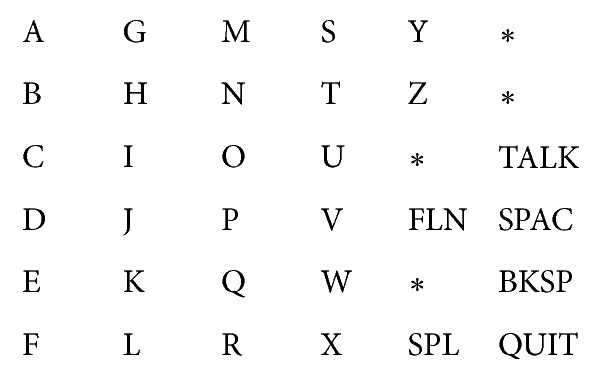
The rows and columns of the matrix were flashed alternately [59].

**Figure 3:**
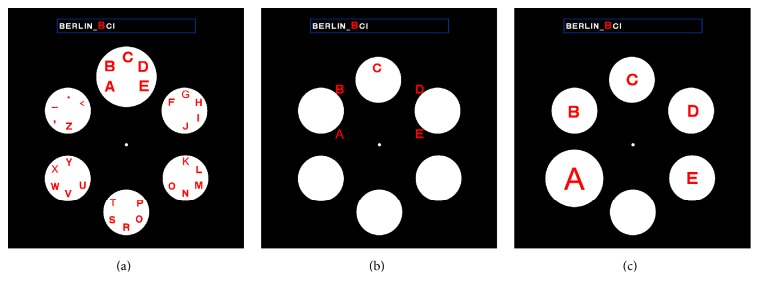
Screenshot of Hex-o-Spell paradigm [60].

**Figure 4:**
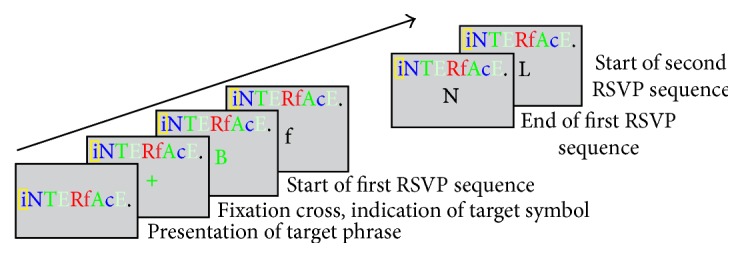
RSVP paradigm [61].

Farwell and Donchin first put forward a P300 speller system [59]: a 6-by-6 matrix containing the letters of the alphabet and a few 1-word commands (see Figure 2) were displayed on a computer-controlled CRT screen. The “stimulus events” that occurred in the test consist of intensifications of either a row or a column of the matrix. The detection was achieved by repeatedly flashing rows and columns of the matrix. When the element containing the chosen character was flashed, a P300 was elicited, and it was this P300 that was detected by the computer. Treder and Blankertz put forward a Hex-o-Spell paradigm and Figure 3 shows a screenshot of the visual speller [60].  Figure 3(a) was the group level. The group containing the target symbol “B” (group “ABCDE”) was intensified.  Figure 3(b) was the transition phase. The symbols of the selected group were expanded onto the other discs.  Figure 3(c) was the symbol level. The nontarget disc with the symbol “A” was intensified. The empty disc at the bottom was intended as a backdoor for returning to the group level in case the wrong group was selected. Acqualagna and Blankertz developed rapid serial visual presentation (RSVP) as a paradigm for mental typewriting for patients unable to overtly fixate the target symbol [61].  Figure 4 showed the process of the paradigm. First, the sentence was presented on the display. After the fixation cross, the RSVP of the symbols starts. The target letter was highlighted on the top of screen. Participants had to concentrate on the target letter and were asked to silently count its number of occurrences. The data recorded in this phase were used to train the classifier. In the online phase, the classifier selected the symbol with the best score and displayed it.

Researchers have recently paid attention to some other ERPs to improve the performance of the ERP-based BCI. Jin et al. use faces as visual stimuli to induce N400 potential to make the ERP more obvious [183, 184]. Jin et al. apply mismatch paradigm to evoke mismatch negativity to improve the accuracy and information transfer rate [185].

### 2.3. MI

#### 2.3.1. Generation Mechanism

Motor imagery may be seen as mental rehearsal of a motor act without any overt motor output. It is broadly accepted that mental imagination of movements involves similar brain regions/functions which are involved in programming and preparing such movements [188]. Pfurtscheller and Neuper showed independent imagination of movements versus planning of voluntary movements of either the right or the left hand; the most prominent EEG changes were localized over the corresponding primary sensorimotor cortex [189]. During the imagination of a right hand or left hand movement, for example, a similar ERD can be found over the contralateral hand area and an ERS over the ipsilateral hand area. Traditionally, transient increases and decreases in spectral power recorded in the human EEG have been termed event-related synchronization (ERS) and desynchronization (ERD), respectively [190]. Both phenomena are time-locked but not phase-locked to the event and they are highly frequency band specific. It has long been known that movements elicit frequency specific changes in the EEG [191–193] and changes in spectral power in the *μ* (8–14 Hz) and *β* (15–30 Hz) frequency bands can be observed during both voluntary [194] and passive movements [195].

During overt execution of the movement, the initially contralateral ERD develops a bilateral distribution [196], whereas during mental simulation this ERD remains mostly limited to the contralateral hemisphere. This means that the suppression of *μ* and central *β* rhythms is more pronounced at the contralateral hemisphere when subjects imagine one-sided hand movements than when they actually perform such movements. These ERD phenomena are used as the classification basis in MI. The most representative MI-ERD phenomena are generated by imaging the movement of left hand, right hand, and feet and are distributed on primary motor cortex (M1). The corresponding areas locate at the EEG electrodes of C3, C4, and Cz which are also the most used electrodes in MI.

#### 2.3.2. Experimental Paradigm

In MI, producing the EEG signal is an important factor in a successful BCI. Therefore, the issues concerned with human training are worth considering. Different from SSVEP or ERP, the MI needs a longer training period in order to generate the ERD/ERS phenomena. It may take months of training before the user achieves the desirable level of performance. In order for the user to acquire self-control of an EEG response, some kind of feedback is essential, at least in the beginning, and the feedback can speed up the learning process and improve performance.

**Figure 5:**
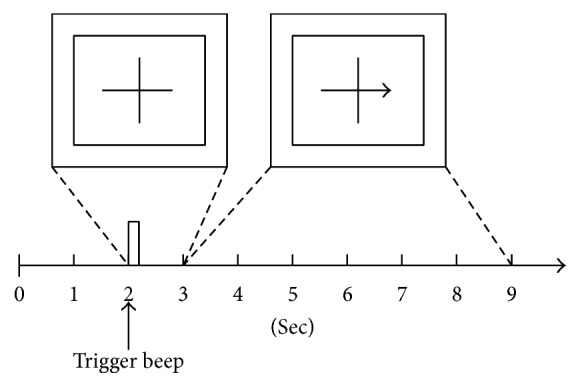
Timing scheme for one training trial [39].

The MI training process usually consists of offline and online training. A period of offline training is essential for adjusting user's EEG signals and training the recognition algorithm. Time for a single training trial is often 9 seconds (see Figure 5). During the trial, an arrow with random direction (left or right) is displayed on the computer screen and the user imagines movement of the left or right hand according to the direction of the arrow. During the first two seconds of one trial, nothing is displayed on computer screen. At *t* = 2 s, a fixation cross appears with a short beep. From *t* = 3 s to 9 s, the user is asked to carry out the MI task while the fixation cross with left or right direction displayed on the screen. Then several trials of training data will be used to generate a template of recognition algorithm. The train generated template is stored to recognize online training. For online training, Neuper et al. used a feedback bar to inform the user of the imaging results [197]. The feedback stimulus began to extend horizontally towards the right or left side according to the classification result. Yu et al. used a hybrid BCI with SSVEP and MI to extend the feedback bar in the targeted direction [198]. Alimardani et al. asked the subjects to watch first-person images of robots through a head mounted display [18]. A lighting ball in front of robot's hands gave motor imagery cue and subjects held images of a grasp for their own corresponding hand. Classifier detected two classes of results (right or left) and sent a motion command to robot's hand.

## 3. Brain Signal Decoding Methods

An essential factor in the successful operation of BCI systems is the methods used to process the brain signals [58]. This paper summarizes different signal processing schemes that have been used in BCI systems. It specifically focuses on the following signal processing components of a BCI: the preprocessing, feature extraction, and feature classification. As for various brain signal evoking mechanisms, this paper chooses the most commonly used paradigms (SSVEP, P300, MI) as the objects to summarize the brain signal processing methods.

### 3.1. Preprocessing Methods

Preprocessing methods in BCI mainly include frequency domain filtering and spatial filtering. Band-pass filters and notch filters are the most commonly used methods in frequency domain filtering, which can extract the characteristic signals located in the stimulus frequency and remove noise and artifacts. These filters are designed according to frequency characteristics of related signals. Often, the frequency range of a band-pass filter is designed according to the stimulation frequencies or their harmonics, while a notch filter is used to remove power line interference. Spatial filters can expand the signal-to-noise ratio of the brain signal response, by processing brain signal data of multiple channels. Signals from multiple channels are less affected by noise than signals from a unipolar or bipolar system. The spatial filtering technique can be also used to extract features. Generally, the spatial filtering methods include minimum energy combination (MEC), canonical correlation analysis (CCA), common average reference (CAR), principal component analysis (PCA), independent component analysis (ICA), and autocorrelation (AC). MEC is used to cancel nuisance signals as much as possible. CCA computes the relation between two multivariable data sets after linear combinations of original data. For the CAR method, the average value of all electrodes is subtracted from the channel of interest to make the EEG recording nearly reference-free. PCA is used to decompose signals into components of responses of brain activities. It aims to reduce the dimension of original data. ICA is often used to separate movement related independent components from EEG data. AC enhances the weak EEG signal, reduces noise, and makes it suitable for analysis.  Table 1 lists some preprocessing methods of different EEG paradigms.

### 3.2. Feature Extraction Methods

The feature extraction is a key issue in signal processing and plays an important role to the whole BCI system. A variety of methods have been used in different EEG paradigms. Several commonly used feature extraction methods are described as follows.

#### 3.2.1. Fourier-Based Transform (FT)

The FT contains Fast Fourier Transform (FFT) and Discrete Fourier Transform (DFT). FT methods are mainly used for power spectrum density analysis (PSDA). FFT is a fast computation algorithm for DFT, which could influence the practical applications. In real applications, the available stimulation frequencies may be limited because the frequency resolutions are limited to a given data segment length. The advantages include simplicity and small computation time.

In SSVEP-based BCI, Wang et al. used 256-point FFT to transform EEG signals into the frequency domain representing 5 frequencies of 9 Hz, 11 Hz, 13 Hz, 15 Hz, and 17 Hz [30]. A 128-point FFT averaged the three spectral components around the target frequency when the subjects did not focus on any stimuli. The average value was used to recognize an idle state of a subject. Mouli et al. used maximum amplitudes of the FFT to distinguish different target stimuli of 7, 8, 9, and 10 Hz [90]. Müller-Putz and Pfurtscheller computed the frequency components by estimating the power density spectrum of the EEG signal with split-radix FFT and averaged the three spectral components around the target frequency [21]. Hwang et al. estimated the EEG data using the FFT with a frequency resolution of 0.1 Hz and constructed the feature vectors by the arithmetic sum of the stimulation frequencies and the second harmonic frequencies [91]. As for DFT, Oikonomou et al. used the FFT algorithm as the estimation of DFT coefficients [92]. Diez et al. also used the FFT as an estimation of the power spectral density based on the DFT [93]. All these studies which used FFT as the estimation of DFT show the computational advantages of FFT. FFT has a wide use in SSVEP systems from low and medium to a high range of frequencies. DFT is often estimated by FFT because of its small computational time.

The P300 components are not sensitive to frequency, so there is no study of it using FFT as feature extraction methods. However, the MI paradigm generates the *μ* and *β* rhythms responses when motor imagery is active. A few studies have tried to recognize the MI tasks using FFT. For example, Hiroyasu et al. used *β* rhythms (13–16 Hz or 13–30 Hz) and *μ* rhythms (8–12 Hz) as the feature values of recognition [111]. The FFT overlap processing was performed to calculate the power spectrum transitions. Jin et al. utilized FFT to analyze the frequency range of *μ* and *β* so as to analyze the energy of EEG and get its features [112].

#### 3.2.2. Wavelet Transform (WT)

EEG signals are nonstationary whose frequency components vary as a function of time [199]. The analysis of such signals can be facilitated by Wavelet Transform which provide flexible time-frequency resolution. WT is based on FT and is an adjustable-window Fourier analysis [200]. The advantage of WT over FT is that it is easy to choose different mother wavelet functions to analyze different types of signals. WT is potentially one of the most powerful signal processing techniques because of its ability to adjust to signal components and its multiresolution which is broadly used to analyze EEG signals.

In SSVEP-based BCI, Zhang et al. introduced Continuous Wavelet Transform (CWT) into SSVEP feature extraction and classification [94]. The choice of mother wavelet is the key issue in CWT. They investigated different types of wavelets and compared the performances in SSVEP classification. Experimental results showed that Complex Morlet wavelet outperformed others and especially had advantages in short EEG data segment. Kumari and Somani used the coefficients of CWT as the feature vectors to find the location of high-frequency components in SSVEP [95].

In P300-based BCI, Demiralp et al. used the WT to identify the most significant response property reflecting the P300 wave [102]. The application of a 5 octave quadratic B-spline-WT on single sweeps yielded discrete coefficients in each octave with an appropriate time resolution for each frequency range. The main feature indicating a P300 response was the positivity of the 4th delta (0.5–4 Hz) coefficient after stimulus onset. Vareka and Mautner applied Daubechies7 wavelet to an averaged target epoch in DWT [103]. The P300 component is obtained by the signal reconstructed from the approximation coefficients of level 6. Guo et al. used Daubechies4 (db4) as the mother wavelet of DWT because of the similarity between db4 and P300 [104]. The decomposition level was set from 4 to 6. They tested the method in traditional P300 speller system. Also, Pan et al. used a WT-based method to recognize P300 components in P300 speller systems [105]. They applied the Mallat algorithm to calculate the coefficients of WT and decomposed the signals into satisfied resolution, which resulted in multiresolution of WT. Vequeira et al. also used WT on P300 speller system as the feature extraction method to help patients with oral communications problems [106].

In MI-based BCI, the CWT gives a highly redundant representation of EEG signals in the time-scale domain [199], so it can be applied for the precise localization of ERD/ERS components in the time-scale domain. Hsu and Sun applied CWT together with Student's two-sample *t*-statistics for 2D time-scale feature extraction [113]. The 2D time-scale yielded a highly redundant representation of EEG signals in the time-frequency domain, from which the precise location of event-related brain desynchronization and synchronization (ERD and ERS) components could be obtained. Then, the CWTs of EEG data performing left and right MI in both C3 and C4 channels were analyzed, respectively. Xu and Song used DWT to execute multiresolution decomposition for a signal [114]. They chose the decomposition level of 4 and the wavelet of Daubechies order 10. The extracted wavelet coefficients showed the distribution of the motor imagery signal in time and frequency and the component D3 (8–16 Hz) was within the *μ* rhythm and D2 (16–32 Hz) was within the *β* rhythm. Bashar et al. proposed Dual Tree Complex Wavelet Transform (DTCWT) domain to identify left and right hand motor imagery movements [89]. DTCWT is a recent enhancement to the DWT which has additional properties including nearly shift invariant and directionally selective of two and higher dimensions [201]. It is more efficient in time-frequency localization of EEG signals. They applied DTCWT to decompose EEG signals into three levels and reconstruct these components using the inverse DTCWT approximately corresponding to the physiological EEG subbands delta, theta, alpha, and beta, respectively. Then, EEG signals in lower frequency bands and *μ* rhythms (7.5–12.5 Hz) were extracted.

According to the references we have consulted, the Wavelet Transform (WT) is suitable for various kinds of EEG paradigms analysis because of its optimal resolution in both the time and frequency domain. Therefore, WT has a wide application in SSVEP, P300, and MI paradigms for feature extraction.

#### 3.2.3. Hilbert-Huang Transform (HHT)

HHT, consisting of empirical mode decomposition (EMD) and Hilbert spectral analysis (HAS) [202], is a recently developed adaptive data analysis method, which has been used extensively in EEG research. The key part of HHT is EMD with which any complicated data set can be decomposed into a finite and often small number of intrinsic mode functions (IMFs). An IMF is an oscillator function with time-varying frequencies that can represent the local characteristics of nonstationary signals [203]. Different from FFT, which is based on cosine functions, HHT is self-adaptive and can acquire better performance in some signal segments, so it can be used in analyzing both stationary and nonstationary signals. However, HHT computation time is higher than that of FT.

In SSVEP-based BCI, Huang et al. used HHT for the recognition of high-frequency SSVEP signals [96]. The original signals were transformed into 11-order IMF, which satisfied the requirements of the HT, by the method of EMD. Then, HT method was used on each order of IMF above to calculate its instantaneous frequency. All those results were used to create an integrated time-frequency figure. The component of its corresponding frequency could be seen from its frequency diagram by analyzing the corresponding levels with FFT. Ruan et al. applied HHT to decompose the independent components by ICA to obtain IMF needed and analyzed IMF by frequency domain analysis or power spectrum estimation [97]. They could identify the subjects at the target stimulus frequency according to the spectrum peak in the spectrum diagram and frequency diagram. Zhang et al. put forward an Improved HHT (IHHT) to extract time-frequency feature of High-Frequency Combination Coding-Based SSVEP (HFCC-SSVEP) [98]. The extraction method consists of synchronous averaging, band-pass filtering, EMD, selection of IMF, instantaneous frequency, and Hilbert spectrum. Besides, the HT has also been employed to compute SSVEP phases [99, 100]. According to the investigation above, HHT provides an effective solution for high-frequency SSVEP.

In P300-based BCI, there is no reference using HHT to extract the P300 components. While in MI-based BCI, HHT is an effective way to extract *μ* and *β* rhythms. Wang et al. used HHT to analyze three motor imagery tasks [115]. The raw signal was decomposed using EMD and several IMFs were gained. Then, the Hilbert spectrum was calculated based on the IMF1 and IMF2. In each motor imagery task, local instantaneous energies, within specific frequency band of electrode C3 and C4, were selected as the features. Jerbic et al. investigated the perspective of HHT for extracting time-frequency information used for MI classification [116]. The IMFs obtained by EMD were mapped into time-frequency-energy matrix, constraining frequency scale to 1 Hz wide frequency bins (range 6–40 Hz). Liu et al. devised a feature, Degree of Imagery (DOI) based on HHT, which can effectively detect the ERD during motor imagery, thereby improving the classification performance [117]. In this paper, they thought that not all of the IMFs were useful for the detection of ERD, so they calculated partial IMFs to accomplish the EMD process in practice in order to improve the computational speed of HHT. Furthermore, they demonstrated that DOI could improve the detection and classification of ERD effect.

The HHT is useful for EEG paradigms that are sensitive to frequency. From the references referred to above, HHT provides an effective solution for high-frequency SSVEP. Also, the *μ* and *β* rhythms in motor imagery can be extracted by HHT.

#### 3.2.4. Independent Component Analysis (ICA)

ICA is a recently developed method with the goal of finding a linear representation of non-Gaussian data so that components are statistically independent or as independent as possible. Such a representation seems to capture the essential structure of the data in many applications, including feature extraction and signal separation [204]. ICA can be performed in two different ways, namely, spatial ICA that extracts unique independent spatial maps and temporal ICA that extracts independent time courses. The electrodes “record” the mixed EEG signal at different locations around the scalp. Therefore, it is reasonable to apply ICA on EEG signals to identify those independent sources and map them to needed components.

In SSVEP-based BCI, ICA is often used to extract EEG signals from raw signals. Wang et al. employed ICA to decompose EEGs over the visual cortex into SSVEP signal and background noise [101]. Thirteen ICs were calculated as sources through ICA and the four with most significant power at stimulation frequency were supposed to be signal activities of SSVEP while the remaining were considered as noise activities.

In P300-based BCI, Li et al. chose FastICA to perform ICA in a P300 speller system because of its fast speed and high reliability [107]. They computed 16 ICs and selected 3 ICs with the largest difference in their coefficients as the P300 related ones. The activation status of these 3 ICs in different channels was used as the feature for P300 identification. Turnip et al. put forward a nonlinear independent components analysis (NICA) extraction method for P300 [108]. With the NICA method, a level of accuracy was attained after about 240 iterations, which were less than 1800 iterations in the same level without using the proposed feature extraction. The results showed that NICA accelerated the network's training process and the tracking error convergence was faster. Li et al. applied ICA to select the channels whose brain signals contained large N200 and P300 potentials and small artifacts as the optimal channels to extract the features [26]. They separated the source signals that produced ERP, muscle artifacts, or ocular artifacts.

In MI-based BCI, Naeem et al. studied three different ICA algorithms (Infomax, FastICA, and SOBI) and compared them to Common Spatial Patterns (CSP), Laplacian derivations, and standard bipolar derivations [118]. Among the ICA algorithms, the best performance was achieved by Infomax when using 22 components as well as for the selected 6 components by visual inspection. Guo et al. explored a dynamic ICA based on the sliding window Infomax algorithm to analyze motor imagery EEG [119]. The method could get a dynamic mixing matrix with the new data input, which was unlike the static mixing matrix in traditional ICA algorithms. The feature patterns were based on the total energy of dynamic mixing matrix coefficients in a certain time window.

In most cases, ICA is used to separate the noise/interference from the raw EEG signals in preprocessing. While in feature extraction, ICA usually combines other feature extraction algorithms to classify the different targets in various EEG paradigms.

#### 3.2.5. Common Spatial Pattern (CSP)

The CSP method is a powerful signal processing technique that was shown to superiorly extract discriminative information, compared to other spatial filters such as bipolar, Laplacian, or CAR [205]. The principle of CSP is yielding a set of spatial filters that are designed to minimize the variance of one class while maximizing it for the other class. Ortner et al. advised that the CSP method needs more electrodes than others [206]. CPS can suppress noise by using the data from many electrodes and hence needs a minimum number of electrodes to perform well. However, because CSP is based on the Fisher discriminative criterion, it can only reflect the separative ability of the mean power of two classes. In practice, this mean power separation may be insufficient to reflect the discrimination of samples around the decision boundary. From the statistics viewpoint, arithmetic mean is sensitive to outliers. Artifacts such as eye and muscle activities may dominate over the EEG signal, and thus they may give excessive power in some channels. Because of CSP simply pooling the covariance matrixes of trials together, if an artifact happens to be unevenly distributed in different experiment conditions, CSP will capture it with high eigenvalue. This will distort the following CSP spatial filter [207].

In P300-based BCI, Pires et al. proposed an application of standard CSP combined with an approach of feature combination based on probabilistic models of spatial filtered data embedded in a Bayesian classifier [109]. The result showed that CSP could be effectively used on P300. Amini et al. used morphological, intelligent segmentation, CSP, and combined features (segmentation+CSP) in the feature extraction block [110]. Within the P300 oddball principle context, they considered two spatiotemporal matrixes which represented the P300 potential evoked by the target event and the ongoing EEG for nontarget events, respectively. Then the set of features was obtained via the CSP technique. A statistical analysis was applied for evaluating the fitness of each feature in discriminating between target and nontarget signals.

Indeed, the CSP is an effective method especially for MI classification. Many improved CSP-based methods have been put forward recently to enhance the classification accuracy. Samek et al. proposed a method called stationary CSP (sCSP) which regularizes the CSP solution towards stationary subspaces; that is, the CSP is extended to be invariant to nonstationarities in the data [120]. CSP reduced variations of the extracted features by assuming that the variations were not task-related like eye movements or electrode artifacts. The results showed that the sCSP was competitive compared with the state-of-the-art CSP method. He et al. proposed an EMD-based CSP method to realize the data-related and adaptive frequency band selection [121]. The IMFs decomposed from the EMD and the amplitude modulated signal by instantaneous amplitude (IA) calculated from HT were fully explored and employed. Use of the EMD filter property avoided manually dividing the frequency band, which was usually adopted in the traditional CSP method. Moreover, it could be expected that a small number of informative frequency band related IMFs would lead to higher algorithm efficiency. To address the problem of selecting the subject-specific frequency band for the CSP algorithm, the Filter Band CSP (FBCSP) algorithm was proposed for MI-BCI. The FBCSP algorithm classifies single-trial EEG based on selected features computed from subject-specific temporal-spatial filters. Keng et al. used FBCSP on BCI competition IV Datasets 2a and 2b to classify 4 classes (left hand, right hand, feet, and tongue) and 2 classes (left hand and right hand) of MI tasks, respectively [122]. Also, Chin et al. used FBCSP to classify 4 classes of MI tasks [123]. To improve the CSP algorithm's robustness against outliers, Yong et al. first investigated how multivariate outliers affected the performance of the CSP algorithm and then proposed a modified version of the algorithm whereby the classical covariance estimates are replaced by the robust covariance estimates obtained using Minimum Covariance Determinant (MCD) estimator [208]. Median Absolute Deviation (MAD) is also used to robustly estimate the variance of the projected EEG signals. The results showed that the proposed algorithm is able to reduce the influence of the outliers. Then, Kai et al. tested the RFBCSP algorithm on BCI competition IV Datasets 2b and the results revealed a promising direction of RFBCSP for robust classifications of EEG measurements in MI-BCI [124].

In the context of Brain Computer Interfaces, the Common Spatial Patterns method is widely used for classification of motor imagery events. However, it is not very often used for classification of event-related potentials such as P300. Meanwhile, there is no reference describing the applications of CSP on SSVEP-based BCI.

All the feature extraction methods we have referred to are most commonly used in BCI, including SSVEP, P300, and MI. Due to article length limitations, we cannot list all the feature extraction methods one by one.  Table 2 summarizes the methods mentioned above in different EEG paradigms.

### 3.3. Feature Classification Methods

Nonstationarities are ubiquitous in EEG signals. They are especially apparent in the use of EEG-based BCI. Therefore the stability of a classifier is a significant factor in the discrimination of targets in various paradigms. Overall, it was agreed that simplicity is generally best and therefore, the use of linear methods is recommended wherever possible. Furthermore, linear classifiers are generally more robust than nonlinear ones. This is because linear classifiers have fewer free parameters to tune and are thus less prone to overfitting. It was also agreed that nonlinear methods in some applications can provide better results, particularly with complex and/or other very large data sets [209].

In the following, the paper introduces the most commonly used classification methods and their applications in BCI systems, which mainly include Linear Discriminant Analysis (LDA), Support Vector Machines (SVM), neural networks, nonlinear Bayesian classifiers, nearest neighbor classifiers, and combinations of classifiers [210].  Table 3 summarizes partial applications of classification methods on SSVEP, P300, MI, and so forth.

#### 3.3.1. LDA (FLDA)

**Figure 6:**
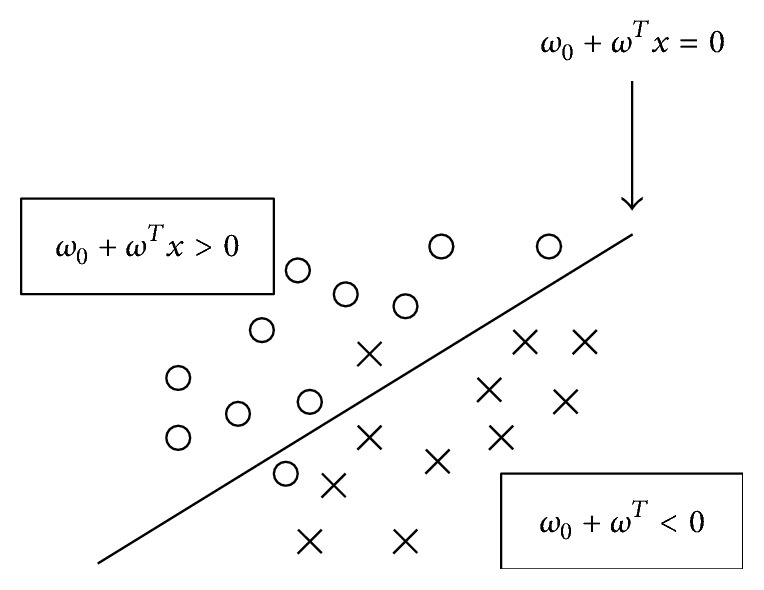
A hyperplane which separates two classes: the “circles” and the “crosses” [186].

The aim of LDA (also known as Fisher's LDA) is to use hyperplanes to separate the data representing the different classes [211]. For a two-class problem, the class of a feature vector depends on which side of the hyperplane the vector is (see Figure 6). LDA finds the optimal projection which maximizes the distance between the two-class means and minimizes the interclass variances. The separating hyperplane is perpendicular to the projection direction [186]. The strategy generally used for multiclass BCI is the “One Versus the Rest” (OVR) strategy which consists in separating each class from all the others.

This technique is simple and has a very low computational requirement, which makes it suitable for online BCI system. Additionally, FLDA is simple to use and generally provides good results. It has been successfully used in a variety of BCI systems. Since the main drawback is its linearity, it may provide poor results on complex nonlinear EEG data. This can be resolved by using a kernel function [212].

To classify the time-varying EEG signals better, an adaptive LDA classifier is needed. Kalman adaptive LDA (KALDA) is an adaptive version of LDA based on Kalman filtering, in which the Kalman gain changes the update coefficient and varies the adaptation speed according to the property of the data [147]. KALDA is a supervised classifier. Maggi et al. put forward a regularized linear discriminant analysis (RLDA) which is based on the modified samples covariance matrix method [127]. The RLDA included a boosting algorithm based on a cyclic minimization of the classification error in the training set and an algorithm for outlier rejection. The multiclass identification problem was solved by means of a combination of binary classifiers using a one-versus-all approach.

#### 3.3.2. Support Vector Machines (SVM)

**Figure 7:**
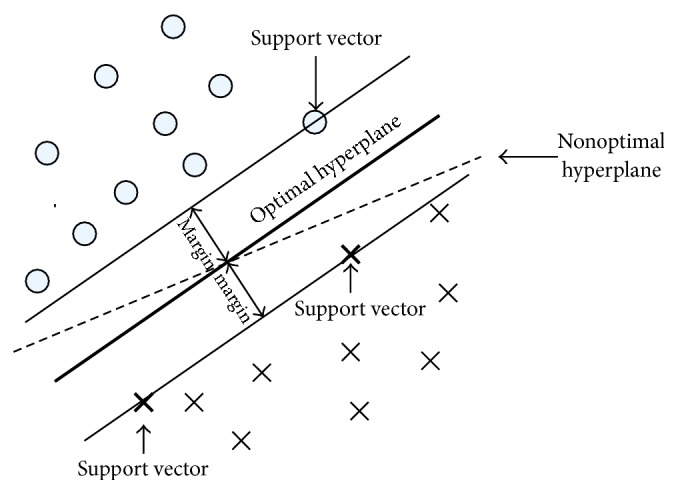
SVM find the optimal hyperplane for generalization [187].

SVMs are becoming popular in a wide variety of biological applications [213]. A SVM is a computer algorithm that learns by example to assign labels to objects. It is also discriminates classes by constructing a linear optimal hyperplane, which is induced from the maximum margin principle between two classes [214]. The selected hyperplane is the one that maximizes the margins, that is, the distance from the nearest training points (see Figure 7). Also, the OVR strategy is used for multiclass BCI.

One of the major advantages of the SVM approach is its flexibility. Using the basic concepts of maximizing margins, duality, and kernels, the paradigm can be adapted to many types of inference problems [187]. Additionally, the usage of SVM is simple. The decision rule of SVM is a simple linear function in the kernel space which makes SVM stable and has a low variance. A low variance may be a key for low classification error in BCI because BCI features are very unstable over time. Furthermore, the robustness of SVM enables SVM to obtain ideal results even with very high dimensional feature vectors and a small training set. However, SVM classifiers have a longer computational time than others.

In order to maintain the classification accuracy and overall performance of the system, online classification and adaptive schemes which modify BCI classification parameters in real-time are particularly important. Jian and Tang applied One Again One Radial Basis Function Support Vector Machine (OAO RBF SVM) to classification in order to improve the short time window classification accuracy [35]. Moreover, they presented a signal quality evaluation method which cancelled the decision of the RBF SVM when signal quality was low and prone to be misclassified. Making no decision could reduce the cost of making a wrong decision. Oskoei et al. applied supervised and unsupervised adaptive schemes to online SVM that classified BCI data [149]. Online SVM processed fresh samples as they came and updated existing support vectors without referring to pervious samples. It was shown that the performance of online SVM was similar to that of the standard SVM, and both supervised and unsupervised schemes improved the classification hit rate. To reduce the time-consuming training sessions, there are also semisupervised SVM learning algorithms. Li et al. designed a Self-Training Semisupervised SVM algorithm for classification in small training data cases [139]. This algorithm converges fast and has low computational burden. They illustrated that the algorithm can be used to significantly reduce training efforts and improve adaptability of a BCI system.

#### 3.3.3. Neural Networks

Neural networks are highly efficient in classification of data and are similar to the working of the human neurons. The method is especially useful when a perfectly algorithmic solution cannot be formulated, but adequate data must be available. Considering these features, a neural network is the best possible solution to classify the BCI. Among all the neural networks used in BCI, the Multilayer Perception (MLP) is the most widely used methods.

MLP is a feedforward artificial NN, in which the Backpropagation (BP) network is the most famous and active model in all the feedforward neural networks. Its kernel is the BP algorithm. BP neural network consists of input layers, hidden layers, and output layers. The number of hidden layers is determined by practical situations. The relationship between the input pattern and the corresponding output pattern can be obtained by learning arithmetic and can be any nonlinear function.

Besides, there are many other neural networks used in the field of BCI, such as Convolutional Neural Network (CNN), CCA-NN, Learning Vector Quantization (LVQ) Neural Network, Multilayer Neural Network (MNN), Adaptive Probabilistic Neural Network (APNN), Time Delay Neural Network (TDNN), and Time-Dependent Neural Networks (TDNN).  Table 3 lists partial practices in different EEG paradigms.

#### 3.3.4. Bayesian Classifiers

The classification principle of the Bayesian classifier is to calculate the posterior probability using Bayesian formulas according to the prior probability of an object, namely, the probability of some class to which the object belongs. The class with the highest posterior probability is the one to which the object belongs. Bayesian classifiers mainly include naïve Bayes classifier, Hidden Markov Model (HMM), and Bayesian Graphical Network (BGN). All these classifiers produce nonlinear decision boundaries. They are generative, which enables them to perform more efficient rejection of uncertain samples than discriminative classifiers. However, Bayesian classifiers are not as widespread as linear classifiers or Neural Networks in BCI applications. The naïve Bayes classifier and HMM have been employed for BCI, but BGN is not commonly used because of its long computational time.

The naïve Bayes classifier greatly simplifies learning by assuming that features are independent given class. Although independence is generally a poor assumption, in practice naïve Bayes often competes well with more sophisticated classifiers [215]. The naïve Bayes classifier is mainly used in motor imagery.

HMMs are very efficient for the classification of time series. They are popular in the field of speech recognition and signal processing, and recently they have been applied to mental task classification of temporal sequences of BCI features and even to the classification of raw EEG. HMMs can also naturally accommodate variable-length models, permit reading of these models, and make sense of them. There are some applications using it in SSVEP, P300, and MI.

#### 3.3.5. Nearest Neighbor Classifiers

These classifiers are very simple. A feature vector is assigned to a class with respect to its nearest neighbor(s). The neighbor can be a feature vector or a class prototype. If the number of samples is large, it makes sense to use it, instead of the single nearest neighbor. The majority vote of the nearest *k* neighbors is called *k* Nearest Neighbor (kNN). kNN is the most widely used classifier among nearest neighbor classifiers.

kNN classifier is rarely applied in SSVEP and P300. However, it has a good performance in MI and has a higher accuracy rate than many other classifiers, such as LDA, Naïve Bayes, and SVM.

Recently, the combination of several classifiers has been employed to solve the feature classifications in BCI. The combination of similar classifiers may outperform the use of the individual classifiers on its own. There are many strategies of classifier combination in BCI applications, such as Boosting [216], Voting [217], and Stacking [218]. Here, we will not explain them in detail. The detailed explanations can be found in the referenced paper [210].

## 4. Typical BRI Systems

### 4.1. Wheelchair Control

As a simple intelligent device, a wheelchair is primarily considered as a BCI-based control object because of its small degree of freedom (DOF). Galán et al. designed an asynchronous and noninvasive EEG-based BCI for continuous mental control of a wheelchair. The subject was able to mentally drive both a real and a simulated wheelchair from a starting point to a goal along a prespecified path by executing three different mental tasks (left hand imagination movement to turn left, rest to go forward, and word association to turn right) [219]. Iturrate et al. used a noninvasive brain-actuated wheelchair that relied on a P300 neurophysiological protocol to realize an autonomous navigation system which drove the wheelchair to the desired location while avoiding collisions with obstacles in the environment detected by the laser scanner [220]. Rebsamen et al. used a slow P300-based BCI to select a destination among a list of predefined locations and a faster MI-based BCI to stop the wheelchair, which provides mobility to BCI users in a safe way [221]. Philips et al. developed an adaptive shared control system of a brain-actuated simulated wheelchair aiming at providing an extra assistance when a subject was in difficult situations. Despite three possible discrete mental steering commands of forward, left, and right, three levels of assistance, including collision avoidance, obstacle avoidance, and orientation recovery, would be triggered whenever the user had difficulties in driving the wheelchair towards the goal [222]. Vanacker et al. introduced a shared control system that helped the subject in driving an intelligent wheelchair with a noninvasive brain interface. The subject's steering intentions were estimated from EEG signals and passed through to the shared control system before being sent to the wheelchair motors [223]. Li et al. proposed a hybrid BCI system combining P300 and SSVEP to improve the performance of asynchronous control and applied the paradigm to produce a “go/stop” command in real-time wheelchair control [224]. In this way, the wheelchair probably plays the role of a human's legs, which guides the disabled or elderly to the place where they want to go.

### 4.2. Manipulator Control

Manipulators mainly refer to a variety of robot arms and mechanical prosthetics. Most of the manipulators have a relatively small DOF, which are able to imitate a human's arm to finish different kinds of tasks. Palankar et al. applied a P300 BCI to control a 7-DOF wheelchair-mounted robotic arm. The BCI interface consists of 15 stimuli corresponding to 14 movements of the robot arm and one stop command, which interpret the user's intention to direct the robot along a step-by-step path to a desired position [225]. Li et al. proposed a BMI system to perform the motion of a serial manipulator in the whole workspace. Small-world neural network (SWNN) was used to classify five brain states based on motor imagery and shared control. The control strategy used six 2-tuple commands to achieve motion control of the manipulator in 3D Cartesian space [226]. Iáñez et al. used four cognitive processes or “tasks” and a rest state to control a robot arm with 6 DOF [227]. Pohlmeyer et al. let a marmoset monkey control the movements of a robot arm for a reaching task using a reinforcement learning (RL) BMI. The monkey was required to move a robot arm to one of two LED targets to receive a food reward [228]. Wang et al. presented a protocol for a three-mode MI-based BCI, in which left/right hand and foot motor imageries were adopted. The three modes constructed eight commands to control a 5-DOF robotic arm to finish “left,” “right,” “up,” “down,” “ahead,” “aback,” “hold,” and “put.” Using the system, the subject was able to move the robotic arm to an appropriate position from the initial position to grab an object, put the object down in a designated position, and move the arm back to the initial position [229]. Elstob and Secco developed a low cost EEG-based BCI prosthetic using MI and realized the open or close of the whole hand by detecting the left or right MI [230]. Müller-Putz and Pfurtscheller used four red LED bars mounted on the hand prosthesis to elicit SSVEP and controlled the prosthesis to finish the tasks of turning right/left and opening/closing hand [21]. Here, controlling a manipulator mainly aims at dealing with some grasping and carrying objects, which takes the place of a human's arms in the BRI system.

### 4.3. Drone Control

Drones are becoming more and more popular in our daily lives. They are widely used in transportation, air shooting, and entertainment. In the application of BRI, Chen et al. established an SSVEP-based BCI system using fuzzy tracking and control algorithm on an air swimmer drone vehicle. The air swimmer drone vehicle was able to elevate, dive, turn left, go forward, and turn right. The system aims at helping subjects with amyotrophic lateral sclerosis (ALS) participate in communication or entertainment [231]. Kos'Myna et al. put forward a bidirectional feedback in MI BCIs, in which the subject was able to control a drone within 5 minutes. They applied the system to the piloting of an AR.Drone 2.0 Quadcopter to do tasks involving taking off, flying in a straight line until a target is reached, and landing the drone [232]. Doud et al. used a MI-based BCI to realize a continuous control of a virtual helicopter through golden rings positioned and oriented randomly throughout a 3D virtual space [233]. In addition, LaFleur et al. realized a quadcopter control in three-dimensional space using a noninvasive MI-based BCI. The subject could pilot the AR Drone Quadcopter safely through suspended-foam rings with the help of the visual feedback of the quadcopter's video on the computer screen [234]. Due to its flexibility and diversity, the drone is a good option for the disabled to communicate with the world.

### 4.4. Humanoid Robot Control

One of the greatest challenges to the BRI systems is the control a humanoid robot, because it has very complex mechanical kinematics and dynamics characters. Bell et al. established an EEG-based BCI interface that can be used to command a partially autonomous humanoid robot to perform complex tasks such as walking to specific locations and picking up desired objects [235]. Li et al. used a 32-channel EEG device to acquire a subject's brainwaves and controlled a humanoid robot, KT-X PC robot, by identifying mental activities when the subject was thinking “turning right,” “turning left,” or “walking forward.” By doing this, they primarily investigated the relationship between complex humanoid robot behaviors and human mental activities [236, 237]. Zhao et al. developed an OpenViBE-based brainwave control system for Cerebot and used the platform to control a humanoid robot NAO to finish four robot-walking behaviors: turning right, turning left, walking forward, and walking backward [27].

In this section, we focus on the development of BRI system from synchronous to asynchronous systems. The controlled objects mainly aim at humanoid robots. Tables 4 and 5 list some BRI applications of controlling humanoid robots with synchronous and asynchronous BCI, respectively.

Table 4 shows that NAO is the most commonly used humanoid robot in BRI systems. There is a wide application for humanoid robots used in BCI including SSVEP, P300, MI, and even their hybrids. Most of them are synchronous systems. Even though the asynchronous BCI systems have been explored a lot in theory, the practical application techniques in social environment are still immature. This is because the detection of idle state is difficult and complex, and the additional classification of idle state is at the cost of accuracy. Therefore, the accuracy of the classification in an asynchronous BRI system often cannot satisfy an operator's demands.

Additionally, the BRI system is still on the level of lab research, and there are few applications currently available. Still, some BRI systems based on BCI have realized online control of intelligent peripherals and feedback. New application systems are emerging continuously. The BRI system has applications in medical and nonmedical fields. In the medical field, patients with a normal functioning mind but a disabled body can use the BRI system to communicate with others and control some intelligent peripherals, such as an intelligent wheelchair, mechanical prosthesis, virtual typewriter, or humanoid robot. While in the nonmedical field, the BRI system can be applied to state supervising of the operator, games, general amusement, and smart homes.

To realize the practical application in daily lives, the safety of the BRI system will be the most significant factor. Considering the safety of the operator, the concept of “brain switch” is put forward. Namely, the brain switch avoids generating task commands in a nontask state, so the brain switch plays an important role in a practical BRI system. For example, when operating a wheelchair or prosthesis, a trigger error may put the operator in danger. The asynchronous BCI system provides a solution by acting as a brain switch. The asynchronous BCI system detects the idle state of brain activities and prevents the output of the control commands while idle. Most BCIs are based on synchronous protocols where the operator must follow a fixed repetitive scheme to switch from one mental task to the next. In these synchronous BCI systems, the EEG recognized phenomena are time-locked to a cue, with a typical trial lasting 4 to 10 s or longer. In contrast, asynchronous BCI relies on asynchronous protocols in which the operator makes voluntary, self-paced decisions on when to stop performing a BCI task and when to start the next one. This makes the system very flexible and natural to operate and yields rapid response times [238].

## 5. Future Perspectives

Over past years, a number of research groups have had success with EEG-based BCI paradigms, including SSVEP, ERP, MI, and their hybrids. Some BRI groups have demonstrated that some BRI systems have the potential for BRI practical applications, such as assisting the elders or disabled persons in daily tasks. However, there are still many technical problems with BCI and BRI that need to be addressed, especially with humanoid robots interaction. In the following, we summarize some difficulties and challenges in future research.

### 5.1. Novel EEG Evoking Patterns

The existing EEG evoking patterns have developed rapidly with respect to principles, coding, and decoding. The classification accuracy has not reached the maturity to control intelligent devices outside a laboratory setting. For example, the visual evoking patterns SSVEP and ERP need visual stimuli equipment, while the MI pattern has the disadvantages of long training time, limited commands, and relatively low classification accuracy. Therefore, novel EEG evoking patterns are essential to begin a new epoch for BCI development. Novel EEG evoking patterns mainly focus on being free of visual stimuli, applying more efficient algorithms to generate more decoding commands, and evoking higher classification accuracy.

### 5.2. Adaptive EEG Decoding Methods

The performance of BCI varies from one person to another and is easily affected by an operator's mental state. To obtain a good performance in the BCI system, the operator must be trained for a while, especially for MI. Therefore, the generality of EEG decoding methods remains unsolved. Considering the similarities and differences among humans, adaptive EEG decoding methods need to be designed so the classification models have a better performance with respect to self-studying and self-correcting. Liu et al. adaptively change repetition number by comparing the classification results with a threshold [239]; Jin et al. detected the same target stimulus twice in limited repetitions, by automatically adjusting the repetition number [240]. In theory, an adaptive classification method plays an important role in online BRI systems.

### 5.3. Portable EEG Device

In a BCI system, the acquisition of the brain signals is the primary function and is the key in guaranteeing the stability and accuracy of the system. With the development of the sensors and amplifiers, the noise attached to the brain signals can be largely restrained. Even though an EEG device has high-precision and high reliability, such as the Cerebus, it is heavy and not portable. Even though the Emotive EPOC is more portable than Cerebus, it has limited channels, which makes it not suitable for multichannel analysis. In the visual evoking paradigms, such as SSVEP or P300, an evoking device is essential, but a LCD screen or a LED device is not suitable for real-world application. A more portable EEG acquisition device is needed and a wearable visual evoking device, such as a Google glass, may solve the problem.

### 5.4. Dynamics and Kinematics and Control Architecture of Robots

In terms of interaction between humans and robots, the dynamics and kinematics of robots are supposed to greatly influence the performance of a BRI system, whether for wheelchairs, manipulator, drones, or humanoid robots. On one hand, the dynamics determines the motion characters, such as the speed, acceleration, and stability. In addition, the dynamics of robots can solve the matching problem between the robot's motion and the information transfer rate (ITR) of BCI. The research of dynamics is used to calculate the time cost of each motion of a robot, which can give guidance for choosing the corresponding ITR. Thus, the entire executing efficiency of a BRI system will be improved greatly. On the other hand, the kinematics of robots plays an important role in path planning, path optimizing, and global path modeling.

A humanoid robot has an especially sophisticated control architecture that consists of sensor fusion, modeling, path planning, and motion control. Solving these problems will greatly prompt the development of BRI in three ways. First, a humanoid robot is generally equipped with different kinds of sensors, such as sonars, cameras, bumpers, and GPS. Taking advantage of the robot's intelligence will assist the operator to finish tasks more efficiently and relieves the mental pressure of the operator. Second, a humanoid robot has a complex mechanical kinematics and dynamics problem, but it can friendly interact with users. Therefore, the application of a humanoid robot in BRI system is becoming more and more popular. Modeling a humanoid robot's mechanical kinematics and dynamics can keep the robot upright walking and assist in path planning and motion control. Third, the former BRI systems mostly control the humanoid robot at a low level and do not combine the operator's intention with the intelligence of the robot for higher level decision making. How and when the brain signals are inserted into the BCI are important considerations for BRI development. For example, path planning can be realized by a camera and GPS, which will never or rarely need the involvement of brain signals. Brain signals only play a role in supervising the process and gives guidance in the case of an emergency. Thus, a human does not need to care about the detailed path a humanoid robot develops but just needs to set a destination. When an emergency occurs, a humanoid robot will be prevented from creating a path and the operator must maneuver via brain signals. Last, there will be conflicts between the user and the robot, so it becomes quite significant to find an appropriate solution to these conflicts. Developing a strategy to find the optimal balance between automation and operator control will be the vital issue in solving the problem.

### 5.5. Evaluation Index System

A system usually needs evaluation indexes to judge its performance. A good evaluation index system should be suitable for different types of systems. For BCI systems, the commonly used evaluation indexes are classification accuracy and ITR. However, both indexes only judge a single experiment of a subject. When conducting the same BCI experiment on the same subject, the indexes must be recalculated. Therefore, the indexes are not adaptive even to the same subject. Average values may solve the problem, but they will cover the differences of the same subject in different spirit status. The evaluation index system of the BCI needs not only classification accuracy and ITR, but also indexes that are able to represent the differences in the same subject. Additionally, the evaluation index system should comprehensively evaluate the entire performance of a BCI system for different subjects.

### 5.6. Individual Differences

The character and amplitude of the brain signal vary from person to person, which leads to the individual differences in the sensitivity and performance of BRI systems. Usually, a person who is familiar with the experimental procedure or has experimental experience will have a high accuracy rate. It is possible for some persons to have a terrible performance in EEG-based BRI systems even after a long training period. Particularly, the MI training process always takes a long time for people to master the skills. Therefore, how to diminish the individual differences between persons still remains to be solved. Additionally, the existing BRI systems mainly use normal functioning people as volunteers, even though there are some applications for special people such as the elderly and patients with neurological conditions. Many experiments are needed to explore the individual differences between a normal functioning person and the disabled or the elderly, for the application of the BRI systems as a service.

### 5.7. Combination of EEG with Other Detecting Means

Despite EEG-based brain signal detecting, there are also many other modern devices capable of detecting a person's brain activity. Some researchers attempted to explore brain activities by combining EEG with functional near infrared spectroscopy (fNIRS) and functional magnetic resonance imaging (fMRI). For instance, Leamy et al. combined fNIRS and EEG to improve motor cortex activity classification during an imagined movement-based task [241]. Putze et al. developed a hybrid BCI which uses EEG and fNIRS to discriminate and detect visual and auditory stimulus processing and found the fusion of the two significantly increased accuracies [242]. Mulert et al. integrated fMRI and EEG to understand brain activities in an auditory oddball paradigm and the results suggest their combination results in an improved understanding of the spatiotemporal dynamics of brain activity [243]. With the emerging of the combination of EEG with other brain signal detecting methods, this technique will be particularly useful in the design of BCI devices and BRI systems.

## Figures and Tables

**Table 1:** Preprocessing methods in different EEG paradigms.

EEG paradigms	Authors	Preprocessing methods
SSVEP	Bevilacqua et al. [66]	2–60 Hz for band-pass filter, notch filter at 50 Hz
	Müller-Putz and Pfurtscheller [21]	0.5–30 Hz for band-pass filter, notch filter at 50 Hz
	Ortner et al. [67]	0.5–100 Hz for band-pass filter, notch filter at 50 Hz
	Wu et al. [65]	0.3–40 Hz for band-pass filter
	Muller et al. [37]	3–60 Hz for band-pass filter, CAR
	Júnior et al. [68]	CCA
	Wang et al. [69]	CCA
	Zhang et al. [70]	Multiset CCA
	Nan et al. [71]	MEC, CCA
	Pouryazdian and Erfanian [72]	PCA

P300	Rakotomamonjy and Guigue [73]	8-order, 0.1–10 Hz band-pass Chebyshev Type I filter
	El Dabbagh and Fakhr [74]	8 order, 0.1–20 Hz band-pass Chebyshev Type I filter
	Mak et al. [75]	0.5–30 Hz band-pass
	Panicker et al. [38]	3 order, 0.5–12 Hz Butterworth filter
	Lugo et al. [76]	0.1–30 Hz band-pass filter
	Lotte et al. [77]	25 Hz low-pass filter
	Li et al. [25]	1–10 Hz band-pass filter
	Spüler et al. [78]	0.5–16 Hz band-pass filter, CAR
	Casagrande et al. [79]	CAR
	Syan and Harnarinesingh [80]	10-order low-pass Hamming-window filter with 6 dB cutoff at 30 Hz, CAR, PCA

MI	Park et al. [81]	5-order, 8–30 Hz Butterworth filter
	Coyle et al. [22]	R^2^CA with a standard 8–26 Hz band
	Wang et al. [82]	FB (Filter Bank) with 4–8,8–12,…, 36–40 Hz
	Devlaminck et al. [83]	A set of spatial filters
	Ang et al. [84]	FB
	Li et al. [85]	8–30 Hz band-pass filter
	Yao et al. [29]	8–26 Hz band-pass filter
	Song et al. [39]	4-order Butterworth IIR filter, Laplacian filter
	Wu and Ge [86]	CAR, FIR (Finite Impulse Response) filter
	Zhou et al. [87]	8–35 Hz band-pass filter, ICA
	Sharma and Baron [88]	PCA, tensor ICA
	Bashar et al. [89]	Autocorrelation

**Table 2:** Feature extraction methods in different EEG paradigms.

EEG paradigms	Authors	Feature extraction methods
SSVEP	Wang et al. [30]	Average and FFT, 5 targets (9, 11, 13, 15, 17 Hz)
	Mouli et al. [90]	FFT, 4 targets (7, 8, 9, 10 Hz)
	Müller-Putz and Pfurtscheller [21]	FFT, 4 targets (6, 7, 8, 13 Hz)
	Hwang et al. [91]	FFT, spelling system (5–7.9 Hz with a span of 0.1 Hz)
	Oikonomou et al. [92]	FFT as an estimation of DFT, 5 target (6.66, 7.5, 8.57, 10, 12 Hz)
	Diez et al. [93]	FFT as an estimation of DFT, 4 targets (37, 38, 39, 40 Hz)
	Zhang et al. [94]	CWT, 4 targets (15, 12, 10, 8.57 Hz)
	Kumari and Somani [95]	CWT, 3 targets (8, 14, 28 Hz)
	Huang et al. [96]	HHT (34, 35, 37, 38, 45, 48 Hz)
	Ruan et al. [97]	HHT (11, 12 Hz)
	Zhang et al. [98]	IHHT (25, 33.33, 40 Hz)
	Molina et al. [99]	HT (all integer frequencies from 30 to 40 Hz, 4 phases)
	Zhu et al. [100]	HT (all integer frequencies from 32 to 40 Hz, 4 phases)
	Wang et al. [101]	ICA (13 Hz)

P300	Demiralp et al. [102]	WT (5 octave quadratic B-spline-WT), auditory oddball paradigm (800, 1200 Hz tones)
	Vareka and Mautner [103]	DWT (Daubechies7), oddball paradigm (traditional OQ experiment)
	Guo et al. [104]	DWT (Daubechies4), P300 speller (6 by 6 matrix)
	Pan et al. [105]	WT (Mallat), P300 Speller (6 by 6 matrix)
	Vequeira et al. [106]	WT (bior), P300 Speller (6 by 6 matrix)
	Li et al. [107]	FastICA, P300 Speller
	Turnip et al. [108]	NICA, EPFL BCI group data
	Li et al. [26]	ICA, oddball paradigm (6 targets)
	Pires et al. [109]	CSP, P300 arrow paradigm
	Amini et al. [110]	morphological, intelligent segmentation, CSP and combined features (segmentation+CSP), P300 Speller

MI	Hiroyasu et al. [111]	FFT, left or right hand (13–16 Hz or 13–30 Hz, 8–12 Hz)
	Jin et al. [112]	FFT, left or right hand (8–30 Hz)
	Hsu and Sun [113]	CWT, left or right hand
	Xu and Song [114]	DWT (Daubechies10), left or right hand
	Bashar et al. [89]	DTCWT, left or right hand
	Wang et al. [115]	HHT, left or right hand, foot
	Jerbic et al. [116]	HHT, left or right hand
	Liu et al. [117]	HHT, left or right hand
	Naeem et al. [118]	ICA, left or right hand, foot, tongue
	Guo and Wu [119]	Dynamic ICA, BCI competition 2003 data set III
	Samek et al. [120]	sCSP, Dataset IVa, BCI Competition III
	He et al. [121]	EMD-based CSP, BCI Competition IV dataset I
	Ang et al. [122] Chin et al. [123]	FBCSP, BCI Competition IV 2a (4 classes) and 2b (2 classes)
	Kai et al. [124]	RFBCSP, BCI Competition IV 2b (2 classes)

**Table 3:** Feature classification methods in different EEG paradigms.

EEG paradigms	Authors	Classification methods
SSVEP	Chu et al. [125]	LDA, 3 classes (20, 15, 12 Hz)
	Bi et al. [126]	LDA, 2 classes (12, 13 Hz)
	Oikonomou et al. [92]	LDA, 5 classes (6.66, 7.5, 8.57, 10, 12 Hz)
	Maggi et al. [127]	RLDA, 5 classes (6, 7, 8, 10 Hz, idle)
	Singa and Haseena [128]	SVM, 4 classes (7, 9, 11, 13 Hz)
	Bi et al. [129]	SVM, 3 classes (12, 13 Hz, idle)
	Sakurada et al. [130]	SVM, 4 classes (6, 7, 8, nonfixation)
	Jian and Tang [35]	OVO RBF SVM, 5 classes (8, 10, 12, 14, 15 Hz)
	Cecotti and Gräser [131]	TDNN, 5 classes (13, 14, 15, 16, 17 Hz)
	Cecotti [132]	CNN, 5 classes (6.66, 7.5, 8.57, 10, 12 Hz)
	Hartmann and Kluge [133]	HMM, 3 classes (10, 12, 15 Hz)
	Ko et al. [134]	kNN, 2 classes (15, 20 Hz)
	Oikonomou et al. [92]	kNN, 5 classes (6.66, 7.5, 8.57, 10, 12 Hz)

P300	Gareis et al. [135]	LDA, P300 Speller
	Onishi and Natsume [136]	Ensemble Stepwise LDA, P300 Speller
	Elwardy et al. [137]	Disjunctive Normal Unsupervised LDA, P300 Speller
	Li et al. [31]	SVM, P300 speller
	Raju et al. [138]	Least Square SVM (LS-SVM), Competition III, Dataset II (P300 Speller)
	Li et al. [139]	Self-Training Semisupervised SVM, P300 Speller
	Yang et al. [140]	LVQNN, 7 classes (oddball paradigm)
	Turnip et al. [141]	MNN, raw data in Hoffmann et al.
	Cecotti and Gräser [142]	CNN, P300 Speller
	Helmy et al. [143]	HMM, raw data in Hoffmann et al.
	Speier et al. [144]	HMM, P300 Speller
	Syan and Harnarinesingh [80]	kNN, P300 Speller, BCI Competition II
	Chikara and Ko [145]	kNN, 2 classes

MI	Chen et al. [32]	LDA, 2 classes (left or right hand)
	Steyrl et al. [146]	Shrinkage RLDA, 2 classes (right hand and feet)
	Vidaurre et al. [147]	KALDA, 2 classes (left or right hand)
	Rathipriya et al. [148]	SVM, 2 classes, Dataset IVa (right hand, foot) and IVb (left hand, foot), BCI Competition III
	Oskoei et al. [149]	supervised and unsupervised SVM, 3 classes, Dataset V, BCI Competition III (left or right hand, word association)
	Siuly and Li [150]	LS-SVM, 2 classes, Dataset IVa and IVb, BCI Competition III
	Hamedi et al. [151]	BP, 3 classes (left or right hand, tongue)
	Wei et al. [152]	LVQNN, 2 classes (left or right hand)
	Hazrati and Erfanian [153]	APNN, 2 classes (left or right hand), BCI competition 2003, data set III
	Haselsteiner and Pfurtscheller [154]	TDNN, 2 classes (left or right hand)
	Siuly et al. [155]	Naïve Bayes, 2 classes, Dataset IVa and IVb, BCI Competition III
	Obermaier et al. [156]	HMM, 2 classes (left or right hand)
	Suk and Lee [157]	HMM, Dataset IIa, BCI Competition IV (2008), 4 classes (left or right hand, feet, tongue)
		kNN, 2 classes (left or right hand),
	Bashar et al. [89]	BCI Competition 2003 data set (motor imagery III)
	Bashar and Bhuiyan [158]	BCI Competition II data set (GRAZ motor imagery III)
	Diana Eva and Tarniceriu [159]	kNN, 2 classes (left or right hand), BCI Competition 2002

**Table 4:** Control of a humanoid robot with synchronous BCI.

EEG paradigms	Authors	Robot model	Control commands
SSVEP	Güneysu and Akin [34]	NAO	Left, right, down, up (hand)
	Zhao et al. [160]	NAO	Turn left, right, walk forward, backward for one-step walking, turn left, right, move forward, stop for continuous walking, head left, right, camera selecting top or bottom, object grasping and lifting
	Caglayan and Arslan [36]	Kondo KHR-3HV	Raise left or right arm
	Zhao et al. [28]	NAO	Walk forward and backward, turning left and right
	Gergondet et al. [23]	HRP-2	Walk forward and backward, turning left and right
	Wang et al. [161]	NAO	Human face detection and tracking

P300	Zhao et al. [28]	NAO	Walk forward and backward, shift left and right, turn left and right
	Li et al. [162]	NAO	Walk forward and backward, shift left and right, turn left and right
	Tang et al. [163]	NAO	Turn left and right (with different angle), move forward (with different speed), stand up, sit down, wave hand, turn on/off the system
	Liu et al. [164]	Adult-size robot	Walk forward and backward, turn left and right

MI	Bouyarmane et al. [165]	Humanoid robot HRP2	Go up and down
	Batula et al. [166]	DARwIn-OP	Walk forward and backward, turn left and right
	Cohen et al. [167]	HOAP3	Walk forward, turn left and right

P300+MI	Finke et al. [19]	Honda's Humanoid Robot	Walk forward and backward, sidestep left and right, turn left and right

SSVEP+MI	Duan et al. [20]	NAO	Walk forward, turn left and right, grasp motion

**Table 5:** Control of a humanoid robot with asynchronous BCI.

EEG paradigms	Authors	Robot model	Control commands
SSVEP	Deng et al. [168]	HanGood HGR-3M	Turn left, right, walk forward, stop

MI	Jiang et al. [169]	NAO	Walk forward, stop, turn left and right
	Jiang et al. [170]	NAO	Stop motion, open/close hand, shoulder up and down, elbow up and down
	Chae et al. [33]	NAO	Head left and right, body left and right, walk forward, stop

SSVEP+P300+MI	Choi and Jo [171]	NAO	Walk forward, body turn, head turn, object recognition
